# Demonstrating a multi-primary high dynamic range display system for vision experiments

**DOI:** 10.1364/JOSAA.384022

**Published:** 2020-04-01

**Authors:** Allie C. Hexley, Ali Özgür Yöntem, Manuel Spitschan, Hannah E. Smithson, Rafal Mantiuk

**Affiliations:** 1 Department of Experimental Psychology, University of Oxford, Oxford, UK; 2 The Department of Computer Science and Technology, University of Cambridge, Cambridge, UK

## Abstract

We describe the design, construction, calibration, and characterization of a multi-primary high dynamic range (MPHDR) display system for use in vision research. The MPHDR display is the first system to our knowledge to allow for spatially controllable, high dynamic range stimulus generation using multiple primaries. We demonstrate the high luminance, high dynamic range, and wide color gamut output of the MPHDR display. During characterization, the MPHDR display achieved a maximum luminance of $3200\;cd/m^{2}$, a maximum contrast range of 3,240,000:1, and an expanded color gamut tailored to dedicated vision research tasks that spans beyond traditional sRGB displays. We discuss how the MPHDR display could be optimized for psychophysical experiments with photoreceptor isolating stimuli achieved through the method of silent substitution. We present an example case of a range of metameric pairs of melanopsin isolating stimuli across different luminance levels, from an available melanopsin contrast of 117% at $75\;cd/m^{2}$ to a melanopsin contrast of 23% at $2000\;cd/m^{2}$.

## 1. INTRODUCTION

The first stage in the visual pathway is the absorption of photons of light within light-sensitive photoreceptor cells. There are five classes of photoreceptors in the human retina that are responsible for the human response to light: the three classes of cones [long-wavelength sensitive (L), medium-wavelength sensitive (M), and short-wavelength sensitive (S)], the rods, and the intrinsically photosensitive retinal ganglion cells (ipRGCs) containing the photopigment melanopsin. To test the contribution of each class of photoreceptor to vision and behavioral responses to light, one would need a method to independently stimulate each photoreceptor class in isolation without interference from the other four photoreceptor classes.

**Fig. 1.**
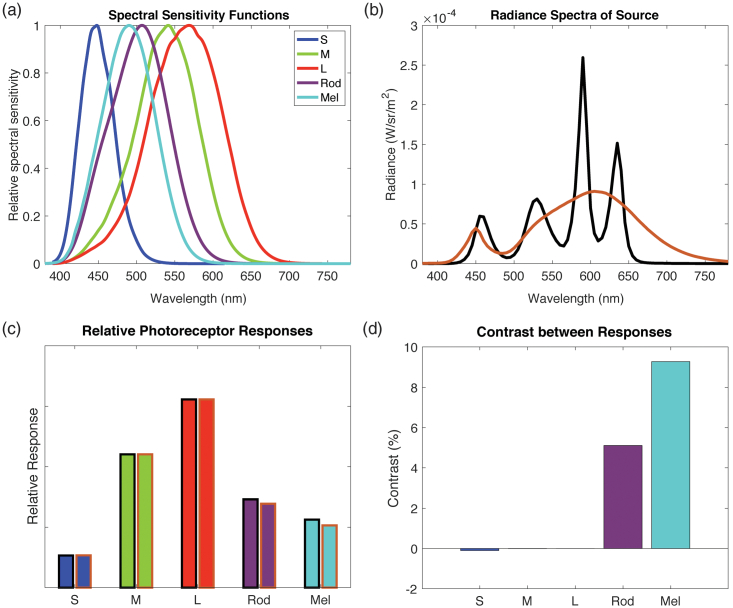
Principle of silent substitution, for the special case of metameric pairs. (a) Spectral sensitivity functions of the photoreceptors. (b) A pair of spectra that are clearly different, yet yield metameric cone responses, with differing rod and melanopsin responses. (c) Relative responses of the photoreceptors to the spectra shown in (b). (d) Percentage difference in response for each photoreceptor type to the spectra shown in (b). The relative activity in the cones is constant, while the relative activity in rods and in melanopsin is different, by 5.1% and 9.3%, respectively. The two spectra in (b) are metamers for cones.

Each photoreceptor class has a certain spectral sensitivity, showing the likelihood of that photoreceptor to respond to a photon of a certain wavelength [1–4]. The spectral sensitivities of all five photoreceptor classes overlap, meaning that it is impossible to find a single wavelength on the visible spectrum that will activate only one of the photoreceptor classes. Such a wavelength would be desirable, as this would be a simple solution to the problem of independent isolation of the photoreceptors. However, one must use an alternative approach to achieve photoreceptor isolation. If we instead consider a stimulus of not just one wavelength, but many wavelengths producing a spectrum of light, we can integrate the spectral sensitivities of the photoreceptors with the spectrum of our stimulus to find the relative responses of the different photoreceptor classes. If we can find two spectra that produce the same response in four of the five photoreceptor classes, but a different response in the fifth photoreceptor class, then we can exchange the two spectra to achieve isolation of an individual photoreceptor class. This is the principle of silent substitution [5]. In the case where we keep the activations of the three cone classes constant, the two spectra are metamers. Without additional constraints, metamers will typically differ in their rod and melanopsin signals (as depicted in Fig. 1).

Independent control of the activations of the five photoreceptors requires control of five independent primary lights. Given control of the intensities, $k_{j}$, of five independent primary lights, $P_{j}(\lambda )$, one can always find a solution for k to produce a specific set of desired photoreceptor activations, $A_{i}$, given the well-established spectral sensitivities of the five photoreceptors, denoted as $R_{L}(\lambda )$, $R_{M}(\lambda )$, $R_{S}(\lambda )$, $R_{R}(\lambda )$, and $R_{I}(\lambda )$, for the L, M, and S cones, the rods, and melanopsin, respectively [Eq. (1)]:

(1) $$k=A\cdot B^{-1},$$

where

$$\begin{array}{ll} k & =\left[\begin{array}{l} k_{1}\\ k_{2}\\ k_{3}\\ k_{4}\\ k_{5} \end{array}\right],\;A=\left[\begin{array}{l} A_{L}\\ A_{M}\\ A_{S}\\ A_{R}\\ A_{I} \end{array}\right],\\ B_{i,j} & =\int_{\lambda =380}^{780}R_{i}(\lambda )P_{j}(\lambda )d\lambda ,\\ & \;i\in \{L,M,S,R,I\},\\ & \;j\in \{1,2,3,4,5\}. \end{array}$$

If one is able to control two spectra, for instance, a test spectrum compared to a reference or background spectrum, one can achieve photoreceptor isolation. Further details on how this can be achieved by the method of silent substitution are described elsewhere [5–7].

Multi-channel Maxwellian-view systems are the most common tool used to achieve independent control of the photoreceptors in practice [8,9]. Such Maxwellian-view systems, however, are limited in their spatial control, and depending on the originating light source may lack intrinsic temporal control. Such Maxwellian-view systems cannot be used to experimentally assess the spatial properties of the photoreceptors and their neural pathways. Projector-based multi-primary display systems allow the experimenter to generate photoreceptor isolating spatially controllable stimuli [10,11]. However, such display systems can currently generate only low luminance, low dynamic range stimuli. If such multi-primary projector-based display systems are used to study the function of individual photoreceptor classes, one cannot assess their function at high luminance levels or over a high dynamic range (HDR). The limitations of current display systems that achieve photoreceptor isolation in practice mean that the experimenter wishing to study the function of individual photoreceptor classes always has to compromise on experimental design.

The multi-primary HDR (MPHDR) display that we present here allows for both spatiotemporal and high luminance HDR control of stimuli to conduct photoreceptor isolating experiments without the compromises imposed by currently available systems. The MPHDR display offers five-independent-parameter spatial control of six effective primaries over a high dynamic range.

### A. Photoreceptor Isolating Experiments Using the MPHDR Display

The MPHDR display enables photoreceptor isolating experiments to be conducted that have not been previously achievable. This opens up an avenue of research on the influences of individual photoreceptors to visual perception and other behavioral responses to light that were previously theorized but lacked the appropriate system for experimental manipulation. Here we discuss some motivating experiments that could be run on the MPHDR display that we present in this paper.

#### 1. Melanopsin

Melanopsin has been recognized as the fifth type of photoreceptor in the human retina, beyond cones and rods, for almost 20 years [12]. Melanopsin is the photopigment found in ipRGCs. ipRGCs are not only intrinsically photosensitive themselves, but also receive strong input from rod and cone pathways [13]. Melanopsin is known to play a role in the control of circadian rhythm [14,15]. Recently, it has been shown that melanopsin is also involved in brightness discrimination [16,17] and the pupillary light reflex [18,19]. Many of the earlier studies into melanopsin function were limited to mouse models where one could knock out subsets of the photoreceptors to isolate the photoreceptor of interest.

Multi-primary displays have enabled the use of silent substitution to study melanopsin mediated visual function independently from rod and cone responses. Four-primary systems allow for the control of melanopsin and the cones [20,21], and with the assumption that rods are saturated at photopic luminance levels, this is sufficient. However, recent work suggests that rods may not be saturated in the photopic range, as discussed in Section 1.A.2. The MPHDR allows for direct control of melanopsin, the cones, and rods. Importantly, the MPHDR enables the study of melanopsin function over a luminance range of up to $2000\;cd/m^{2}$, which was previously unachievable in multi-primary systems, and which is of particular interest for melanopsin research given the unusual adaptation characteristics of ipRGCs and their role in mediating responses to environmental light levels [13].

In the first instance, the MPHDR could be used to replicate the results of studies showing the contribution of melanopsin to pupil control. One method to test this using the capabilities of the MPHDR would be to produce metamers, differing in melanopsin content, across a range of luminance levels and measure pupillary response to a high versus low melanopsin contrast stimuli at each luminance level. One would anticipate greater pupil constriction with the high melanopsin contrast stimuli than the low melanopsin contrast stimuli at each luminance level, and a general overall constriction of the pupil at higher luminance levels. Through an experiment such as this, one can truly isolate the influence of melanopsin on the pupil response while controlling for the confounding influence of luminance.

#### 2. Cones and Rods

While the contributions of the rods and cones to vision have been arguably easier to study experimentally than melanopsin, there is still a large scope for psychophysical studies of rods and cones beyond the cathode ray tube (CRT) gamut. This is a niche that has traditionally been occupied by Maxwellian-view systems using combinations of narrowband lights. However, these systems offer less flexibility in spatial control.

One common technique for assessing cone contribution to vision is by performing experiments in the photopic range, where rods are believed to be saturated [22]. However, recent work in mice suggests that rods may actually still influence visual perception and cone activity at high luminance levels, previously considered to be within the range of rod saturation [23]. Previous studies have considered methods for investigating rod and cone interactions using four primary photostimulators to independently control cone and rod contrast [20,21]. Using the MPHDR display, one could investigate the influence of rod activity on cone pathways and vice versa at a range of luminance levels and also investigate the spatial properties of such an interaction.

The MPHDR is not limited to psychophysical studies of rod, cone, and melanopsin activity, but can also be used to investigate post-receptoral processing of these signals.

## 2. METHODS

### A. Hardware Design

The hardware system design is inspired by earlier work that was based on combination of a digital light processing (DLP) projector and a liquid crystal display (LCD) [24]. The DLP projector was used to replace the static backlight of a commercial LCD monitor such that the backlight was dynamically controlled to create a dual-modulation display system. In the MPHDR setup, two DLP projectors introduce a way to modify the primary colors of this backlight, and updated components improve the image quality with respect to the original design. Moreover, we further explain the custom modifications that must be made to the individual components in order to construct the system. The main components of the hardware are given in Table 1.

**Table 1.** Components of the Display System

Component	Brand	Properties	Modification	Acronym
LCD panel	LP097QX1 (No brand or manufacturer information available)	2048×1536 IPS 9.7 in.	Reflective layer removed	LCD
Projectors	Acer x128H (Acer UK Ltd, Greater London, UK)	1024×768	Color wheel removed	$P_{1}$, $P_{2}$
Fresnel lens	Comar Optics (Comar Optics Ltd, Cambridge, UK)	Focal length 305 mm	NA	FL
Diffusers	Rosco (Rosco, London, UK)	3028 1/4 tough white diffusion	NA	$D_{1}$
		3040 powder frost	NA	$D_{2}$
Filters	Thorlabs (Thorlabs LTD., Ely, UK)	NF514	NA	$F_{1}$
		BF500	NA	$F_{2}$
		NF514	NA	$F_{3}$
		NF488	NA	$F_{4}$

**Fig. 2.**
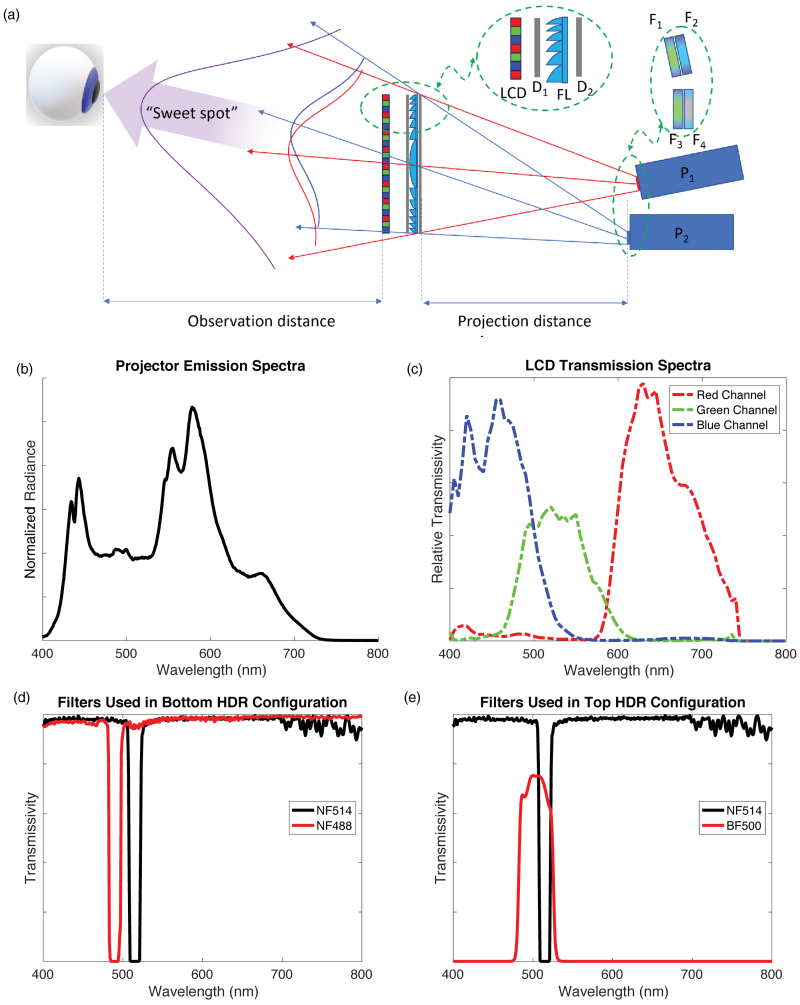
(a) Schematic of the display setup. Observers should view the display from the “sweet spot,” the optimal position for overlap of the backlight from each projector. The schematic depicts the light path from the two DLPs (P1 and P2), passing through the filters stacked in front on the DLPs (F1–F4) through two diffusers (D1 and D2) which sandwich a Fresnel lens (FL) before passing through the LCD panel (LCD) and reaching the observer. (b) Relative emission spectra of the projector lamp after passing through the DLP chips used in the display (without filters). (c) Transmissitivity of the LCD panel used in the display, measured before construction of the setup. The spectra of the DLPs and LCD transmission were measured using a JETI Specbos 1211. (d) Transmission of the filters used in the bottom HDR configuration, where the bottom projector ($P_{2}$) illuminates the LCD. (e) Transmission of the filters used in the top HDR configuration, where the top projector ($P_{1}$) illuminates the LCD. The transmission of the filters reported here is specification data provided by the manufacturer, ThorLabs [25,26–27].

In the MPHDR setup, depicted in Fig. 2(a), the two projectors increase the color gamut and increase the number of controllable primaries. The backlight is produced by two independent projectors that have different filters. The filters were selected to provide maximum available melanopsin contrast and maximum available color gamut. The spectra of the individual components used in the setup are depicted in Figs. 2(b)–2(e).

#### 1. Modification of the LCD Panel

**Fig. 3.**
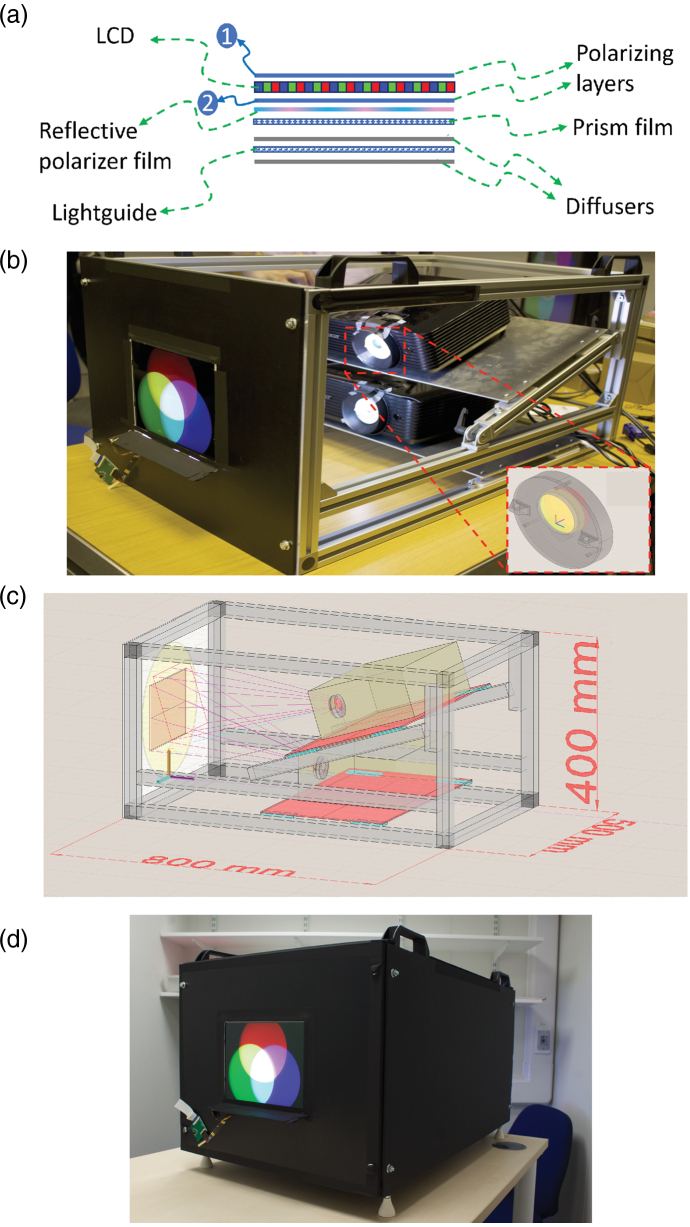
(a) Layers of a generic LCD panel. The layer labeled 1 indicates the front polarizing layer of the LCD, and the layer labeled 2 indicates the back polarizing layer of the LCD. These were the only two additional layers of the LCD panel, along with the LCD itself, kept in the final display setup. (b) Photograph of the MPHDR without the final casing. The CAD design of one of the 3D printed filter mounts is shown as an inset. (c) Representative CAD drawing of the setup. (d) Photograph of the MPHDR with final casing.

The first step is to modify the most delicate component in the system, which is the LCD panel. In the MPHDR setup, we used the high-resolution LCD display panel given in Table 1. Modern LCD panels contain several light enhancing layers, which are stacked to improve the light efficiency of the display [28]. These layers are used to improve brightness and screen uniformity by recycling the light generated in the backlight. We removed all the layers, which are depicted in Fig. 3(a), to reach the bare LCD panel. In our configuration, we placed the LCD panel such that the front side of the LCD panel [shown as 1 in Fig. 3(a)] is directed towards the projector. This is because the front side of the panel contains an anti-reflective treatment, which helps to reduce the reflections of the DLP projection, which is acting as a backlight. The light throughput was significantly higher when placing the LCD panel in this orientation.

#### 2. Modification of the DLP Projectors

To maximize the brightness of each DLP projector, we removed the color wheel from the light path. We had to retain the color wheel inside the projector, as the control circuits prevented the projector from operating when the color wheel was disconnected. After the color wheel was removed, the exposed empty space was covered with a custom-made 3D-printed part to prevent stray light inside the projector. Then, the emission spectra were modified by adding custom filters in front of the lens to improve the available gamut and melanopsin contrast. Custom-designed 3D-printed mounts to hold the filters in place at the exit aperture of the projector lens were attached, as shown in the inset of Fig. 3(b). The key difficulty was in the design of the 3D-printed parts so that they were not exposed to the direct beam path coming from the lamp. This was because the temperatures produced by the beam are significantly above the thermal limits of materials used for 3D printing.

#### 3. Choosing the Appropriate Filters

The display was designed with independent control of melanopsin in mind, as the first experiments we plan to run on our system will be using the method of silent substitution to modulate melanopsin stimulation. While appropriate for isolating any of the five human photoreceptor classes, the filters in our setup, Figs. 2(d) and 2(e), were chosen to maximize melanopsin contrast and color gamut. If the goal was instead to isolate S cones via silent substitution, then one may wish to select filters such that S cone contrast and color gamut were maximized.

By measuring the DLP emission spectra and the transmissivity of the LCD panel [Figs. 2(b) and 2(c)], one can predict the effective primaries in the display, as each effective primary is a simple multiplication of the relevant DLP emission spectrum with the relevant LCD transmission spectrum. Using the display algorithm described in Section 2.C, it is then possible to predict the spectral output of the display and calculate the available contrast of each photoreceptor and available gamut. Without filtering the light from the DLPs so that the two DLPs have different spectra, the display would simply be a typical three-primary HDR. By filtering the light, one is able to achieve the multi-primary control described here.

To choose the appropriate filters for our setup, we performed an “n choose k” search of all possible combinations of appropriate commercially available filters. Due to the physical positioning of the filters in front of the aperture of the DLPs, the filters had to be at least 25 mm in diameter so as not to clip a significant amount of the light emitted from the DLP. We compiled a non-exhaustive list of commercially available filters that fit this description. We then modeled the emission spectrum for each DLP with any combination of two, one, or none of the commercially available filters filtering the DLP emission spectrum. A combination search was performed on this space, to pick the two filter combinations that were appropriate for the two DLPs to provide spectra with maximum melanopsin contrast and maximum available gamut.

#### 4. Optical System Design

Most projectors have an off-axis image formation; hence, the light rays leave the objective lens above the optical axis. This has implications for the design of the filter mount and the design of the light frustum. We assumed the light rays were leaving a rectangular area on the lens surface and designed the light frustum between the lens and the image on the screen accordingly.

In order to direct the light towards the observer, improve display uniformity and efficiency, we put a diffuser screen (Rosco, London, UK) and a Fresnel lens (f=305mm, Comar Optics, Cambridge, UK) in front of the modified LCD panel. However, when we placed a diffuser before the Fresnel lens (i.e., when we realize the image from the projector on the diffuser first), the transmitted light reflected between the LCD panel and the lens surface, since both surfaces are reflective. Therefore, we added a second diffuser after the Fresnel lens. This also improved the visual quality by reducing the visibility of rings on the Fresnel lens, thereby making the illumination more uniform. The diffusers were stretched and fixed such that the gaps between the diffusers and the Fresnel lens were minimal, especially on the side with the lens grooves. We also chose the focal length of the lens so as to form converging light rays towards the observer.

The final display configuration can be thought of as two HDR configurations stacked on top of each other. We refer to the HDR configuration driven by the top projector [shown as $P_{1}$ in Fig. 2(a)] as the top HDR configuration and to the HDR configuration driven by the bottom projector [shown as $P_{2}$ in Fig. 2(a)] as the bottom HDR configuration. For the top HDR configuration, in which the top projector illuminates the LCD, we aligned the projected image according to the LCD position. We considered three options for creating overlapping images from the two HDR configurations:

1. **Beam-splitter option**. We considered a 90° rotated projector, projecting images from an axis folded 90° relative to the main projector optical axis on the lateral plane. Although this would allow optimal alignment and calibration, this configuration suffers from light loss due to the beam splitter.
2. **Flipped top projector option**. We considered rotating the top projector 180° around its own optical projection axis creating a mirror symmetry of the bottom projector with respect to the center of the LCD panel. Although this design makes it easier for mechanical construction of the system, it suffers from significant divergence of light from the two projectors. In this arrangement, someone viewing the display from the center of the LCD, would receive significantly lower light from either the bottom or the top projector, as the main beam paths would be above or below the central axis, respectively.
3. **Slanted top projector option**. Ultimately, the top projector was mounted at an angle above the bottom one, aiming the beam towards the center of the LCD as shown in Fig. 3(c). We found this configuration the most convenient since the physical alignments were easier and the light loss was minimal. The beam paths were close to one another such that when the display is viewed from the center of the LCD, it is possible to receive significant amounts of light from both projectors, as in Fig. 2(a). The top projector was positioned such that the image on the diffuser overlapped with the image projected from the bottom projector. The tilt angle of the top projector was measured as approximately 25°. Therefore, the overlapping region of the images, i.e., “sweet spot” in Fig. 2(a), was defined within this angle. The size and the focusing of the image was adjusted accordingly. Two drawbacks of this configuration were the keystoning correction requirement and the reduced light levels due to the off-axis positioning of the top projector with respect to the bottom projector.

The entire system was enclosed to prevent stray light reaching the viewing position [Fig. 3(d)]. In order to maintain stable running conditions, we installed two inlets and two exhaust fans for the enclosure, as the projectors produce a large amount of heat.

### B. Calibration

The MPHDR is calibrated in multiple steps. The calibration procedure is repeated for the two HDR configurations: the top HDR configuration and the bottom HDR configuration. In the first step, we find the geometric transformation from DLP pixel coordinates to the LCD pixel coordinates, so that we can align DLP and LCD pixels. This is achieved by displaying a grid of points first on the LCD, then on the DLP, capturing both with a camera (Canon 550D, Canon Inc., Tokyo, Japan), and finding both homographic transformation and mesh-based warping to obtain high-quality alignment of the two displays. Next, a grid of points on the DLP is captured to measure and model the amount of blurring introduced on the DLP by the diffuser and due to its lower resolution. Then, an image of a uniform white field is taken and used to compensate for the spatial non-uniformity of the display. Because the slight vignetting caused by the filters on the DLP projectors prevented detection of the corners of the calibration grid, the filters were removed during geometric calibration. The keystoning of both projectors was first reduced using projector adjustments, and then the final alignment was achieved using our geometric calibration procedure.

It is important to note the difference in the resolution reproduced by the LCD panel and the DLP projectors. The spatial resolution of the DLP projectors was half the resolution of the LCD panel (refer to Table 1). In addition to that, the projected DLP images were blurred by the diffuser. The resulting blur was well approximated by a radially symmetric Gaussian function with the σ=18.35 [LCD pixels] for the bottom projector and σ=9.78 [LCD pixels] for the top projector. We compensated for the blur of the DLPs by sharpening the image on the LCD panel.

Once the camera-based calibration is complete, the second step of the calibration procedure, the linearization, follows. In linearization, the display response curves (analogous to a gamma curve for CRTs) for both DLPs and the LCD panel are measured in turn using a JETI Specbos 1211 (JETI Technische Instrumente GmbH, Jena, Germany). To measure the display response curve of the LCD panel, the bottom HDR configuration is switched on to its maximum output, while the top HDR configuration is switched off. Display response curves are measured for each of the individual LCD channels, red, green, and blue, in turn. The display response curve is then measured with all three of the LCD channels simultaneously as a linearity check. The display response curve for each DLP is measured in turn, by setting the LCD panel to white (equal settings for red, green, and blue) at an appropriate transmissivity to measure the DLP display response curve, while switching off the other DLP.

The third and final step of calibration, spectroradiometric calibration follows. Spectroradiometric calibration was performed using a JETI Specbos 1211 (JETI Technische Instrumente GmbH, Jena, Germany) or Cambridge Research Systems SpectroCAL MKII Spectroradiometer (Cambridge Research Systems, Kent, UK). The spectroradiometric calibration measures the spectrum of each effective primary in turn, with the display set to an appropriate intensity to allow reliable measures of the spectrum of each effective primary (see Section 2.C). We verified our spectroradiometric calibrations using a SpectraScan PR-670 spectroradiometer (Photo Research, Syracuse, NY).

For all calibration steps, the calibration device (i.e., the camera for the camera-based calibration or the spectroradiometer for the linearization and spectroradiometric calibration), is placed at the “sweet spot” for the MPHDR display. This means that the calibration device is aligned at the point of maximal overlap of the primaries from the top HDR configuration and the primaries from the bottom HDR configuration. The calibration device is at a height defined by the sweet spot and a distance of 45 cm from the LCD panel. The device is oriented so its optical axis is perpendicular to the LCD panel.

### C. Display Algorithm

To drive the display, we consider the control the user would want over the stimulus. To design cone, rod, and melanopsin isolating stimuli, the experimenter would want to know, at each spatial location, the full spectrum the observer sees when viewing the display.

The spectral output of the MPHDR, s(λ), can be thought of as the spectral emission from each DLP, $E_{1}(\lambda ),E_{2}(\lambda )$, passed through the LCD with a certain transmission spectrum for each of the three channels in the LCD, $T_{R}(\lambda ),T_{G}(\lambda ),T_{B}(\lambda )$, as in Eq. (3).

For each spatial location, the experimenter has control of five independent parameters of the MPHDR display: the intensity of each of the DLPs, $k_{E_{1}}$, and $k_{E_{2}}$; and the intensity of each channel of the LCD, $k_{T_{R}}$, $k_{T_{G}}$, and $k_{T_{B}}$, forming the parameter vector κ as in Eq. (2):

(2) $$\kappa =[k_{E_{1}},k_{E_{2}},k_{T_{R}},k_{T_{G}},k_{T_{B}}].$$

The value of all of these parameters can vary from near zero to one. These parameters can never be zero, as this would result in a display with infinite contrast, which is not possible with this type of display configuration.

The spectral output at each spatial location of the MPHDR is thus

(3) $$s(\lambda )=\sum_{i}\sum_{j}k_{E_{i}}k_{T_{j}}E_{i}(\lambda )T_{j}(\lambda ),$$

where

i∈{1,2},j∈{R,G,B}.

If one could measure the spectral transmission of each of the channels of the LCD and the emission spectra of the projectors, then one could generate the output spectra. However, due to physical limitations of the display, it is tricky to get a reliable measure of these spectra, $E_{i}(\lambda )$ and $T_{j}(\lambda )$. What we can easily and reliably measure are the effective primaries we get as the output of the display, when the emission spectra of the projectors have passed through the spectral transmission of the LCD panel. We can consider six effective primaries, $P_{n}$, from the display, as shown in Eq. (4):

(4) $$P_{n}(\lambda )=E_{i}(\lambda )\cdot T_{j}(\lambda ).$$

**Fig. 4.**
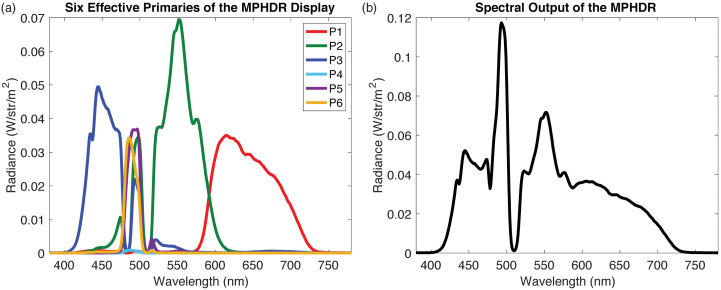
(a) Six effective primaries available from the MPHDR display. Note that the spectra of primaries $P_{4}(\lambda )$, $P_{5}(\lambda )$, and $P_{6}(\lambda )$ are measured from 380 nm to 560 nm given the transmission properties of the bandpass cut-off filter placed in the top HDR configuration. (b) Spectral output of the MPHDR display when all six effective primaries are set to maximum.

Therefore, written out in full form in Eq. (5), the output spectra from the display can be modeled as

(5) $$\begin{array}{ll} s(\lambda ) & =k_{E_{1}}\left(k_{T_{R}}P_{1}(\lambda )+k_{T_{G}}P_{2}(\lambda )+k_{T_{B}}P_{3}(\lambda )\right)\\ & \;+k_{E_{2}}\left(k_{T_{R}}P_{4}(\lambda )+k_{T_{G}}P_{5}(\lambda )+k_{T_{B}}P_{6}(\lambda )\right), \end{array}$$

where, for example, one can measure $P_{1}(\lambda )$ by setting $k_{T_{R}}$, $k_{E_{1}}=1$ and $k_{T_{G}}$, $k_{T_{B}}$, $k_{E_{2}}$ to zero. In practice, the measurement of $P_{1}(\lambda )$ will include some small contribution from the black levels of the remaining LCD channels and DLP, as the minimum achievable setting for any of the constants, $k_{n}$, is 0.0005 in the current MPHDR configuration. Analogous settings can be chosen to measure $P_{2}(\lambda )$, $P_{3}(\lambda )$, $P_{4}(\lambda )$, $P_{5}(\lambda )$, and $P_{6}(\lambda )$, resulting in a space where the user has six effective primaries [as shown in Fig. 4(a)] that he/she can control through five independent parameters to generate the spectral output from the MPHDR display [Fig. 4(b)].

The code to run the MPHDR display uses custom-built drivers written in OpenGL (Khronos Group, Beaverton, OR, USA) and MATLAB (MathWorks, Natick, MA, USA). The experimenter can program an experiment using Psychtoolbox [29], or other appropriate libraries, and simply render individually to each of the three components of the MPHDR. The experimenter will specify a pattern with colors defined as [$k_{T_{R}}$, $k_{T_{G}}$, $k_{T_{B}}$] to send to the LCD screen, a pattern with colors defined as [$k_{E_{1}}$, $k_{E_{1}}$, $k_{E_{1}}$] to send to $DLP_{1}$, and a pattern with colors defined as [$k_{E_{2}}$, $k_{E_{2}}$, $k_{E_{2}}$] to send to $DLP_{2}$. The parameters $k_{T_{R}},k_{T_{G}},k_{T_{B}},k_{E_{1}},k_{E_{2}}$ are relative linear color values.

### D. Designing Photoreceptor Isolating Stimuli

To generate pairs of photoreceptor isolating stimuli, we need to adjust the parameters of the MPHDR display system. Given the six effective primaries of the MPHDR, one cannot find a unique solution for the intensities of the individual primaries to design photoreceptor spectral pairs as in Eq. (1). This therefore becomes an optimization problem. Since the six primaries are controlled by five parameters, we seek two parameter vectors, $\kappa_{1}$ and $\kappa_{2}$. These refer to five specific settings for the parameters described in the section above. These two parameter vectors are defined as in Eq. (6):

$$\kappa_{1}=[{k_{E_{1}}}_{;1},{k_{E_{2}}}_{;1},{k_{T_{R}}}_{;1},{k_{T_{G}}}_{;1},{k_{T_{B}}}_{;1}],$$

and

(6) $$\kappa_{2}=[{k_{E_{1}}}_{;2},{k_{E_{2}}}_{;2},{k_{T_{R}}}_{;2},{k_{T_{G}}}_{;2},{k_{T_{B}}}_{;2}],$$

producing a photoreceptor-selective, or “isolating” spectral pair, $s_{1}(\lambda )$ and $s_{2}(\lambda )$.

The defining characteristic of this spectral pair is that when alternating between them, the activation of only one photoreceptor class is changing (the “isolated” class), while the activation of the other photoreceptor classes (the “silent” classes) stays the same. The spectral sensitivities of the human photoreceptors are well established, and we denote them as $R_{L}(\lambda )$, $R_{M}(\lambda )$, $R_{S}(\lambda )$, $R_{R}(\lambda )$, and $R_{I}(\lambda )$ for the L, M, and S cones, the rods, and melanopsin, respectively.

The activation of a photoreceptor, R(λ), to a given spectrum, s(λ), is given as in Eq. (7):

(7) $$A=\int_{\lambda =380}^{780}R(\lambda )s(\lambda )d\lambda .$$

We calculate the Weber contrast for a given photoreceptor class as the percent difference in activation experienced under $s_{1}(\lambda )$ and $s_{2}(\lambda )$. One of these spectra serves as the background spectrum, $s_{1}(\lambda )$, giving activation $A_{1}$, and the other as the modulation spectrum, giving activation $A_{2}$. The Weber contrast between background and modulation, i.e., the percent difference in activation experienced under $s_{1}(\lambda )$ and $s_{2}(\lambda )$, is defined as in Eq. (8):

(8) $$C=\frac{A_{2}-A_{1}}{A_{1}}.$$

The two spectra are selected so that the contrast of the isolated photoreceptors is $C_{isolated}>0$, and the contrast of the silent photoreceptors is $C_{silent}=0$.

For example, for a melanopsin isolating modulation: $C_{I}>0$, and $C_{L}=C_{M}=C_{S}=C_{R}=0$. In practice, it is useful to allow for some tolerance and accept modulations that are $C_{I}>0$, and $C_{L}<\eta$, $C_{M}<\eta$, $C_{S}<\eta$, $C_{R}<\eta$, where η is an acceptable “splatter” contrast. Alternatively, another metric, such as Euclidean pooled cone contrast could be subject to a constraint: $\sqrt{C_{L}^{2}+C_{M}^{2}+C_{S}^{2}}=0$ for the exact case or $\sqrt{C_{L}^{2}+C_{M}^{2}+C_{S}^{2}}<\eta$ for the “tolerant” case. For modulations that are cone-silent, instead of optimization in the photoreceptor space, one can also optimize in a derived space, such as the *xyz* coordinates calculated from the LMS cone activations, or some other color space.

**Fig. 5.**
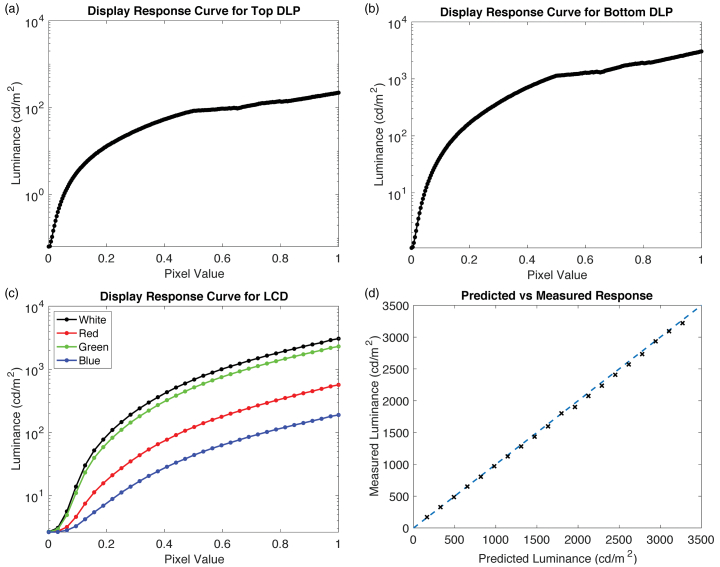
(a) Display response curve for the top projector. (b) Display response curve for the bottom projector. (c) Display response curve for the LCD panel. (d) Predicted and measured luminance (black cross symbols) matches after display response correction.

Here, we express the relationship between background and modulation as the difference between the log transformed activations [Eq. (9)]:

(9) $$\log A_{2}-\log A_{1}.$$

To find photoreceptor isolating modulations, we optimize over $\kappa_{1}$ and $\kappa_{2}$ to maximize the quantity in Eq. (10):

(10) $$\log(A_{2;isolated})-\log(A_{1;isolated}).$$

Our optimization can then be described as

(11) $$\underset{\kappa_{1},\kappa_{2}}{argmax}\log(A_{2;isolated})-\log(A_{1;isolated}).$$

The optimization is subject to the constraint in Eq. (12):

(12) $${\left(\log(A_{2;silent})-\log(A_{1;silent})\right)}^{2}=0,$$

or for the soft constraint version [Eq. (13)],

(13) $${\left(\log(A_{2;silent})-\log(A_{1;silent})\right)}^{2}<\epsilon .$$

The above procedure does not constrain luminance or chromaticity of the background and/or modulation spectra but simply produces a spectral pair with the largest contrast on the isolated photoreceptors. In situations where it is desirable to fix the background activations at some desired level, both the optimization target and the constraint can be written as the difference to the desired set of activations.

## 3. RESULTS

Here we present measurements taken from the MPHDR display that demonstrate the key benefits of the display over existing display systems and the ability of the MPHDR display to generate photoreceptor isolating stimuli. Measurements were taken using either the Cambridge Research Systems SpectroCAL MKII Spectroradiometer, the JETI Specbos 1211, or the SpectraScan PR-670.

### A. High Dynamic Range and High Luminance Output

Typical CRT monitors may reach a maximum luminance of approximately $100\;cd/m^{2}$, while typical LCD monitors may reach a maximum luminance of approximately $300\;cd/m^{2}$. The MPHDR display can reach a maximum luminance of $3200\;cd/m^{2}$. The luminance contribution from each of the HDR configurations in our setup is not identical, with the bottom HDR configuration contributing a maximum luminance of $3000\;cd/m^{2}$ and the top HDR configuration contributing a maximum luminance output of $200\;cd/m^{2}$. Both projectors and the LCD are subject to display response correction (analogous to gamma correction for CRTs), as shown in Figs. 5(a)–5(c). After display response correction, the MPHDR behaves linearly [Fig. 5(d)].

The full on/off contrast range and ANSI contrast range of each of the individual components (the LCD, and each DLP) and configurations (each HDR configuration, and the full MPHDR setup) of the MPHDR display were calculated by measuring the maximum and minimum luminance, $L_{\text{max}}$ and $L_{\text{min}}$, of the LCD, T, and each DLP, $E_{1}$ and $E_{2}$, in turn. The full on/off contrast range is the dynamic range achievable from a uniform black screen to a uniform white screen, while the ANSI contrast range is the dynamic range achievable in practice when a 2×2 white and black checkerboard pattern is displayed. Both the full on/off and ANSI contrast range, D, for each component and configuration can be calculated as described in Eq. (14):

(14) $$D=\frac{L_{T_{\text{max}}}}{L_{T_{\text{min}}}}\cdot \frac{\left(L_{E_{1_{\text{max}}}}+L_{E_{2_{\text{max}}}}\right)}{\left(L_{E_{1_{\text{min}}}}+L_{E_{2_{\text{min}}}}\right)},$$

where the choice of the parameter vectors, κ, for the luminance measures are as follows:

$$\begin{array}{ll} L_{T_{\text{max}}}:\kappa & =[0,0,1,1,1],\\ L_{E_{1_{\text{max}}}}:\kappa & =[1,0,0,0,0],\\ L_{E_{2_{\text{max}}}}:\kappa & =[0,1,0,0,0],\\ L_{T_{\text{min}}}:\kappa & =[0,0,0.0005,0.0005,0.0005],\\ L_{E_{1_{\text{min}}}}:\kappa & =[0.0005,0,0,0,0],\\ L_{E_{2_{\text{min}}}}:\kappa & =[0,0.0005,0,0,0]. \end{array}$$

For the individual components, one can simply calculate the dynamic range as maximum measured luminance divided by minimum measured luminance from the component. For the HDR configurations, Eq. (14) can be applied where for the individual HDR configurations the luminance of the DLP not driving that configuration is simply zero. Both the full on/off and ANSI contrast ranges of the individual components and configurations and the full MPHDR display are reported in Table 2. The MPHDR display has a full on/off contrast range of 3,240,000:1 and an ANSI contrast range of 341,000:1.

**Table 2.** Comparison of the Full On/Off (Global) and ANSI (Local) Contrast Range of the Individual Components and Configurations of the MPHDR Display Compared to the Full MPHDR Display Configuration

Display	Full On/Off (Global) Contrast	ANSI (Local) Contrast
LCD panel	1140:1	725:1
Top DLP	3430:1	100:1
Bottom DLP	2810:1	594:1
Top HDR configuration	3,930,000:1	72,400:1
Bottom HDR configuration	3,220,000:1	431,000:1
MPHDR	3,240,000:1	341,000:1

### B. Expanded Color Gamut

**Fig. 6.**
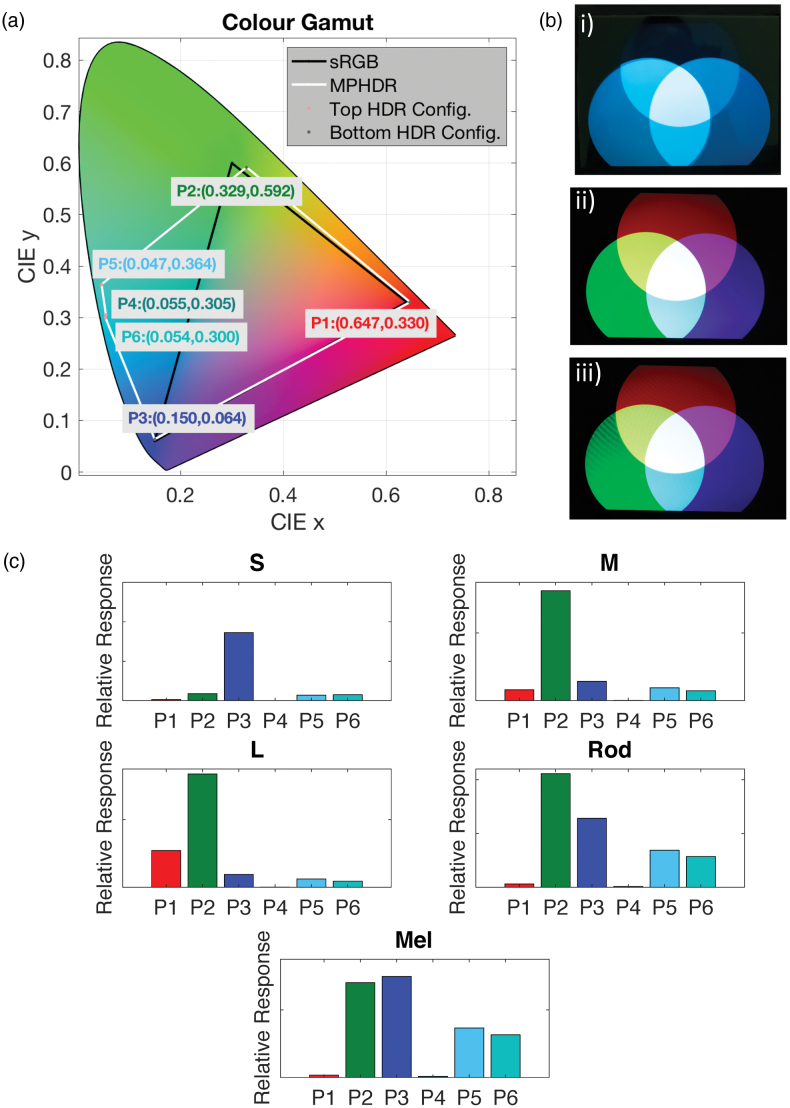
(a) Color gamut available from the MPHDR display, broken down into the two HDR configurations when all six effective primaries are available, expressed using the CIE 1931 xy chromaticity coordinates. The sRGB gamut is shown for comparison. (b) Photographs of the display when (i) only the top HDR configuration is on (for the circles top, bottom-left, and bottom right, respectively: κ=[1,0,1,0,0], κ=[1,0,0,1,0], κ=[1,0,0,0,1]); (ii) only the bottom HDR configuration is on (κ=[0,1,1,0,0], κ=[0,1,0,1,0], κ=[0,1,0,0,1]); (iii) MPHDR when both HDR configurations are contributing (κ=[1,1,1,0,0], κ=[1,1,0,1,0], κ=[1,1,0,0,1]). Note that the fringes seen in (b.iii) have been introduced by the camera’s optics and are not visible on the display. (c) Bar charts showing the relative response of each photoreceptor type to each primary, appropriately scaled for each photoreceptor class.

**Fig. 7.**
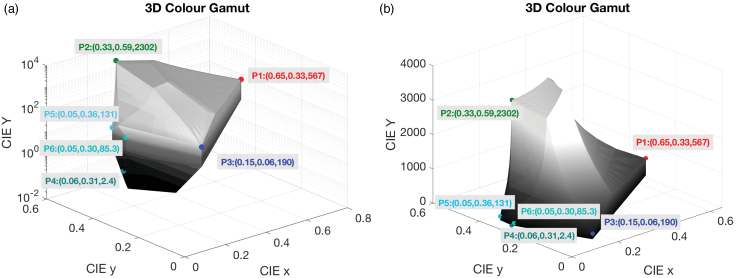
(a) 3D color gamut in CIE xyY chromaticity coordinates, plotted in log scale for the luminance axis. (b) 3D color gamut as in (a), shown again here in CIE xyY chromaticity coordinates, in linear scale for the luminance axis. These figures are also provided as movie files in Visualization 1 and Visualization 2.

The maximum color gamut available from the MPHDR display is wider than the color gamut provided by traditional sRGB displays shown in the CIE xy chromaticity diagram in Fig. 6(a). The two HDR configurations also contribute differently to the gamut due to the filters chosen for the DLPs, depicted in Figs. 6(a) and 6(b). To further visualize the expansion of the gamut, Fig. 7 shows the full gamut the MPHDR is able to achieve in CIE xyY chromaticity space, in both log scale [Fig. 7(a)] and linear scale [Fig. 7(b)] for luminance. These figures are also provided as movie files in Visualization 1 and Visualization 2 to view the full gamut in CIE xyY chromaticity space from different views of the 3D plot. Visualizing the gamut in CIE xyY chromaticity space is useful to see how the available gamut changes as a function of luminance.

Using the CIE xy and CIE xyY chromaticity diagrams is a helpful tool in understanding the expanded color gamut available from the MPHDR display, but it does not capture the full expansion of the gamut, as the CIE xy diagram is limited to L, M, and S cone responses. To truly gauge the expansion of the gamut, one needs to consider the expansion in rod and melanopsin space as well as cone space. Bar charts, showing the relative responses of each photoreceptor type, when each primary in turn is set to maximum while all other primaries are switched off are shown in Fig. 6(c), to more completely visualize the expanded gamut of the MPHDR display. The primaries $P_{4}(\lambda )$, $P_{5}(\lambda )$, and $P_{6}(\lambda )$ in particular allow additional modulation of rod and melanopsin responses.

### C. High Contrast Photoreceptor Isolating Stimuli

Using the algorithm described in Sections 2.C and 2.D, we are able to produce photoreceptor isolating stimuli with the display. We consider the example of designing melanopsin isolating stimuli, as the filters used to filter the spectra of the DLPs were chosen to optimize the available melanopsin contrast and gamut, meaning that in its current form, the MPHDR display is best suited to melanopsin isolating experiments.

**Fig. 8.**
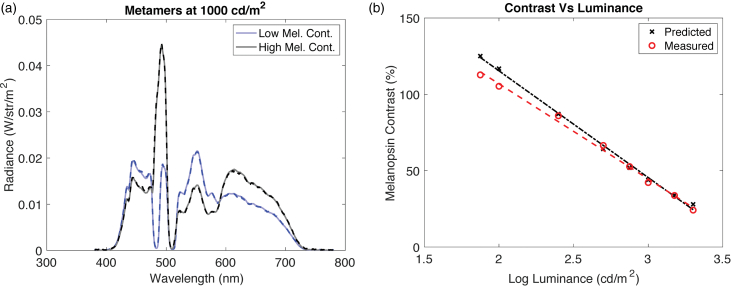
(a) Example of a metameric pair of melanopsin isolating stimuli. The measured spectral output from the MPHDR display (shown as a dashed line) overlaps the desired spectral output from the MPHDR display (shown as a solid line) generated using the display algorithm. (b) Available melanopsin contrast as a function of luminance, for both the measured contrast and predicted contrast.

The MPHDR is able to generate metameric pairs of melanopsin isolating stimuli such that the cones are silenced while the relative response of melanopsin to the two spectra differs. The spectra of the desired metamers can be displayed reliably on the MPHDR. An example case demonstrating this is shown in Fig. 8(a). In this instance, we ignore rod activations.

We are able to achieve a maximum melanopsin contrast of 117% at a luminance of $75\;cd/m^{2}$. Available melanopsin contrast decreases exponentially as a function of luminance, as depicted in Fig. 8(b). Even at a high luminance level of $2000\;cd/m^{2}$, we are still able to achieve a melanopsin contrast of 23%. While we ask for cone silencing (i.e., cone contrasts of 0%) when designing our metamers, in practice we find small cone signals due to measurement uncertainty in the display calibration. At luminance levels between $75\;cd/m^{2}$ and $100\;cd/m^{2}$, our metamers (at maximum melanopsin contrast) produce 7% cone contrast; at luminance levels between $500\;cd/m^{2}$ and $1500\;cd/m^{2}$, the cone contrasts are <1%, and at other available luminance levels, the cone contrasts are <3%. If the experimenter wished to completely silence the cones, or to generate metamers at lower luminance levels for specific stimuli, the spectral output of the display could be fine-tuned.

## 4. DISCUSSION

The goal of vision research is to understand how the human visual system responds to and integrates visual stimuli. To do so, vision researchers need a method to generate controlled and adjustable stimuli. Three common features of a stimulus that the experimenter may want to control are the spatiotemporal properties of the stimulus, the luminance and/or contrast range of the stimulus, and the spectral content of the stimulus. Before the implementation of the MPHDR display, there was no display system that could give the experimenter complete control of all three of these features, meaning the experimenter always had to compromise.

For instance, to ask questions about the spatial resolution of the visual system, one needs to use a display that offers spatial control, such as a conventional CRT or LCD monitor [30]. Such displays also offer intrinsic temporal control of the stimulus at the frame rate. However, a CRT or an LCD monitor would not be appropriate for an experiment aiming to probe the visual system across the full range of photopic light levels, and one may need to opt for a Maxwellian-view setup for such an experiment [31]. Multi-channel Maxwellian-view systems can also allow the experimenter control of more than three primaries, and could be used to achieve photoreceptor isolation, something that conventional three-primary displays such as CRTs cannot achieve [8,9]. Such Maxwellian-view systems, however, are limited in their spatiotemporal control. Multi-primary spatially controllable stimuli are achievable through projector-based display systems, but such systems can generate only low dynamic range stimuli [10,11]. Development of HDR display systems has improved the ability of vision researchers to conduct experiments at high luminance levels and high contrast in a spatially controllable display; however, existing systems of this type have been limited to only three primaries [24].

The limitation of existing display systems means that the vision researcher has always had to make a choice: spatiotemporal control, HDR, multi-primary; pick up to two. The MPHDR display that we demonstrate is the first system, to our knowledge, that offers the vision researcher control of all three desirable controls of a stimulus, as summarized in Table 3.

**Table 3.** Comparison of Features of the Different Systems Available for Vision Experiments^*a*^

Display	High Dynamic Range	Spatiotemporal Control	>3 Primaries
CRT monitors	✗	✓	✗
LCD monitors	✗	✓	✗
HDR displays	✓	✓	✗
Multi-primary Maxwellian view systems	✓	✗	✓
Multi-primary projector-based displays	✗	✓	✓
Multi-primary high dynamic range display	✓	✓	✓

^*a*^ The MPHDR display is the only system to our knowledge to incorporate all three key features: high dynamic range, spatiotemporal control, and more than three primaries.

The HDR of the new display comes from replacement of the standard backlight from an LCD panel with a DLP projector, as described in earlier HDR systems [24]. In addition to expanding the dynamic range, allowing us to achieve a full on/off contrast ratio of 3,240,000:1 and an ANSI contrast range of 341,000:1, this setup also allows for extremely bright displays, with the MPHDR display reaching a peak luminance output of $3200\;cd/m^{2}$. The benefit of the HDR component of the MPHDR display is that it allows stimuli to be presented with a dynamic range and absolute light level that is closer to the range seen in real-world environments.

The multi-primary nature of the new display comes from adding in a second HDR configuration to the traditional HDR setup. The benefit of the multi-primary component of the MPHDR display is twofold. First, it allows for a wider color gamut than traditional displays. Second, (and crucially) it allows for independent control of the photoreceptors on a spatially controllable HDR system, creating a new branch of possible experiments on the function of individual photoreceptor classes.

The benefit of the design of the MPHDR display described in this paper is that it is affordable and achievable. Any vision researcher who wishes to build such a display for his/her own experiments should be able to follow the approach outlined above and construct the MPHDR display using off-the-shelf components.

## 5. FUTURE DEVELOPMENT

The model for driving the MPHDR takes as input the five parameters that the experimenter can control, namely, the intensities of each DLP and the intensities of each channel of the LCD, and gives a spectral output, which can be considered as the addition of the six effective primaries of the MPHDR, as specified in Eq. (5). One may note the fact that we do not have access to as many controllable independent parameters as we have effective primaries. This is due to the limiting factor of the LCD color channels. Once we have set a value for say the red channel of the LCD, we are constraining the range of settings for the “red” primary from both the top and bottom HDR configurations. The only further control we have of the value for these two red primaries is the intensity from each DLP.

To move towards an MPHDR with fully independent control of the six output primaries, we consider the following alternative approaches:

1. **Temporal blurring**. One solution would be to modulate which DLP is illuminating the LCD at any given time. Currently, the LCD has a frame rate of 60 Hz. If we turned only one of the DLPs on for one frame of the LCD to produce three independent output primaries, and then turned on the other DLP during the following frame, we could change the LCD settings between the two frames to produce an additional three independent output primaries. This would reduce the effective frame rate of the display to 30 Hz, as pairs of frames would be treated as one temporally blurred frame. To ensure synchronicity between the DLPs and LCD, and to ensure sharp transitions between the two DLP illuminations, we would opt for a shutter device to control the on/off switch of the DLPs. A 30 Hz refresh rate would be sufficient for some vision science experiments, but would be limiting for others.
2. **Monochromatic LCD and color DLPs**. Another alternative approach for fully independent six-primary control would require a fundamental change to the hardware design presented above. In our setup, the color wheels of the DLPs have been removed, rendering the spectra from the projectors spatially uniform, while the LCD has chromatic transmission filters, namely, the RGB channels. If a setup instead kept the color wheels of the DLPs intact and projected through a monochromatic LCD, one could have independent control of the chromatic properties via the DLPs instead of the LCD. The concern for this option is that the color wheels cause significant light loss in the DLP, so one would need an expensive high-end DLP to achieve the HDR and luminance output that is possible with the current approach.
3. **Custom designs**. A custom fully independent MPHDR display could be developed by creating an LCD panel with four or more sub-pixels; by displaying multiple backlight colors in a single frame [32]; or by adding color channels to HDR light steering projectors [33]. Such displays, although feasible, would require custom manufacturing and substantial engineering effort. This is in contrast to the MPHDR design we demonstrate, which can be built from inexpensive off-the-shelf components and with limited engineering experience.

For the purpose of running photoreceptor isolating experiments on the display via silent substitution, our five-parameter control of the spectral output of the MPHDR is sufficient. It is nonetheless interesting to think about how one would develop a fully independent six-primary MPHDR display.

## 6. CONCLUSION

We present, to our knowledge, the first implementation of a MPHDR system for use in vision experiments. The MPHDR display has five independent parameters that allow for the control of six effective primaries from the display. During the characterization of the MPHDR display, we achieved a maximum luminance output of $3200\;cd/m^{2}$, a full on/off contrast of 3,240,000:1, an ANSI contrast of 341,000:1, and an expanded color gamut. We believe that this system will be useful for many different psychophysical experiments, particularly for modulation of individual photoreceptor classes. As an example case, we generated a range of metameric pairs of melanopsin isolating stimuli across different luminance levels, from an available melanopsin contrast of 117% at $75\;cd/m^{2}$ to a melanopsin contrast of 23% at $2000\;cd/m^{2}$. We also envisage that new schemes in display architectures (such as temporal blurring and monochromatic pixelated active masks with colored backlighting) will enable full independent control of multiple primaries in a HDR display.
